# Superiority of Trans-Oral over Trans-Nasal Sampling in Detecting *Streptococcus pneumoniae* Colonization in Adults

**DOI:** 10.1371/journal.pone.0060520

**Published:** 2013-03-28

**Authors:** Krzysztof Trzciński, Debby Bogaert, Anne Wyllie, Mei Ling J. N. Chu, Arie van der Ende, Jacob P. Bruin, Germie van den Dobbelsteen, Reinier H. Veenhoven, Elisabeth A. M. Sanders

**Affiliations:** 1 Department of Pediatric Immunology and Infectious Diseases, Wilhelmina's Children Hospital, University Medical Centre Utrecht, Utrecht, The Netherlands; 2 Department of Medical Microbiology and The Netherlands Reference Laboratory for Bacterial Meningitis, Academic Medical Centre, Amsterdam, The Netherlands; 3 Regional Laboratory of Public Health, Haarlem, The Netherlands; 4 Unit Vaccinology, Centre for Infectious Disease Control, The Netherlands Vaccine Institute, Bilthoven, The Netherlands; 5 Linnaeus Institute, Spaarne Hospital, Hoofddorp, The Netherlands; University of Malaya, Malaysia

## Abstract

The human nasopharynx is the main reservoir for *Streptococcus pneumoniae*. We applied conventional and molecular methods to determine the prevalence of *S. pneumoniae* nasopharyngeal colonization in adults. Paired trans-orally and trans-nasally obtained nasopharyngeal samples from 268 parents of 24-month-old children were assessed for pneumococcal presence. Parents were classified as colonized when live pneumococci were recovered from either sample cultured on medium selective for *S. pneumoniae*. Of the 52 (19%) colonized parents 49 (18%) were culture-positive in trans-nasal and 10 (4%) in trans-oral samples. Bacterial growth was harvested from these cultures, DNA isolated and tested by quantitative-PCR (qPCR) targeting *lytA* and *piaA* genes specific for *S. pneumoniae*. A sample was considered positive if signals for both genes were detected. Altogether 105 (39%) individuals were classified as positive for pneumococcus by qPCR including 50 (19%) in trans-nasal and 94 (35%) in trans-oral settings. Although significantly more trans-nasal compared to trans-oral samples were culture-positive for *S. pneumoniae* at the primary diagnostic step (p<0.001) the opposite was observed in qPCR results (p<0.001). To confirm the presence of live pneumococcus in samples positive by qPCR but negative at the initial diagnostic step, we serially-diluted cell harvests, re-cultured and carefully examined for *S. pneumoniae* presence. Live pneumococci were recovered from an additional 43 parents including 42 positive in trans-oral and 4 in trans-nasal samples increasing the number of individuals culture- and qPCR-positive to 93 (35%) and positive by either of two methods to 107 (40%). There were significantly more trans-oral than trans-nasal samples positive for pneumococcus by both culture and qPCR (n = 71; 27%; vs. n = 50; 19%; p<0.05). Our data suggest that pneumococcal colonization is more common in adults than previously estimated and point towards the superiority of a trans-oral over a trans-nasal approach when testing adults for colonization with *S. pneumoniae*.

## Introduction

*Streptococcus pneumoniae* is a commensal of the human upper respiratory tract and an important pathogen causing a range of bacterial diseases from mild and common respiratory infections of childhood like otitis media, to potentially fatal pneumococcal disease like pneumonia or meningitis. Since pneumococcal colonization is considered a necessary step prior to infection [1] there is growing interest in studies focusing on the dynamics of pneumococcal carriage. In general, colonization rates are assumed to be highest in young children and to decline with age, resulting in pneumococcal carriage in elderly being scarcely detected [2]–[6]. This shift seems mainly attributed to shorter colonization episodes and lower exposure with age [7]–[10]. Variation in colonization rates within a given age-group seems to be largely determined by patterns of social contacts. Besides situations of crowding such as in day-care centers, intra-family transmission is considered one of the most important sources of new acquisition [9], [11]. As a result, siblings and parents of very young children are more likely to carry pneumococci compared to the general population, contributing to the spread of the pathogen within the community [4], [11]–[13]. In order to better understand the factors affecting *S. pneumoniae* transmission and host-pathogen interactions during asymptomatic colonization, infection and invasive disease, accurate assessment of colonization patterns, incidence rates and carriage duration is important for all age groups. This information is also important for the accurate measurement of the impact of the pneumococcal vaccines or any other preventive strategies.

Currently, the gold standard for detection of pneumococcal carriage is the isolation of live pneumococci from bacteriological cultures of trans-nasally collected nasopharyngeal swabs [14]. This approach seems to work well for very young children because the nasopharynx is considered to be relatively easy to access via the trans-nasal route. In adults the nasopharyngeal niche seems less accessible and alternative approaches are also explored. This includes sampling of the oropharynx [12], [15], [16] or the posterior wall of the nasopharynx approached trans-orally [17], [18]. Since there is no clear evidence that the sensitivity of either of these sampling methods surpasses the other and usually only either the trans-nasal or the trans-oral sample is culture-positive for pneumococci, the accepted solution in adults is to obtain and process both trans-nasal and trans-oral nasopharyngeal samples simultaneously [3], [17]–[20].

In this study we applied a quantitative-PCR (qPCR) approach [21] to assess the potential increase in sensitivity of pneumococci detection in trans-nasally and trans-orally obtained nasopharyngeal samples. For this purpose, we collected paired samples from parents of 24-month-old children that were screened for carriage of common respiratory pathogens as part of a national surveillance study. To increase the chances of identifying the presence of pneumococci by qPCR, we processed harvests of pneumococci-selective culture plates, a step that was introduced as an equivalent of culture-enrichments advocated by others [15], [22], [23]. The results of our molecular assay were compared with direct isolation of live pneumococci from culture.

The first key finding of this study was the high rate of pneumococcal presence detected in healthy asymptomatic adults by both culture and qPCR. This suggests that rates of *S. pneumoniae* carriage in mature human hosts may be largely underestimated. The second key finding was the supremacy of trans-oral over trans-nasal swabs in the detection of pneumococcal carriage in adults when molecular methods were applied.

## Materials and Methods

### Ethics Statement

The study was approved by an acknowledged Dutch National Ethics Committee (Stichting Therapeutische Evaluatie Geneesmiddelen, http://www.stegmetc.org). Written informed consent was obtained from all individuals and the procedures followed were in accordance with European Statements for Good Clinical Practice and the declaration of Helsinki of the World Medical Association.

### Streptococcus pneumoniae carriage study

Trans-nasal and trans-oral nasopharyngeal swabs were collected from 268 parents of asymptomatic 24-month-old children during a pneumococcal surveillance study performed in the Netherlands in the fall/winter season of 2010/2011. Samples were collected from one parent per family. Deep trans-nasal nasopharyngeal samples were obtained by trained personnel using flexible sterile Copan E-swabs (Copan, Brescia, Italy) according to World Health Organization standard procedures [14]. Trans-oral samples were collected with rigid sterile Copan E-swabs under direct observation of the posterior pharynx [19]. Samples analyzed here were collected in the study on long term effects of heptavalent pneumococcal conjugate vaccine on nasopharyngeal carriage of selected bacterial respiratory pathogens in vaccinated children and their parents. A detailed description of the study population and primary results were recently published by Spijkerman *et al*. [24].

### Isolation of *Streptococcus pneumoniae* by conventional culture approach

All samples were transferred in liquid Amies medium (Copan) to the Regional Laboratory of Public Health in Haarlem and cultured within 24 hours on trypticase soy agar supplemented with 7% defibrinated sheep blood plus gentamicin 5 mg/l (gentamicin plates, SB7-GENT medium, Oxoid, Badhoevedorp, the Netherlands). Gentamicin plates were inoculated with Amies medium using the original swab and incubated overnight at 35°C and 5% CO_2_. Cultures were processed for *S. pneumoniae* by a conventional diagnostic approach: isolation of a single alpha-hemolytic colony susceptible to optochine and soluble in deoxycholate [25]. A person was considered colonized when live pneumococci were recovered from any sample tested.

### Secondary use of primary cultures

Primary cultures on gentamicin plates were stored at room temperature until being transferred to the research laboratory of the UMC Utrecht for further processing. On arrival the cultures were harvested by washing the plate with 2 ml of Brain Heart Infusion (Oxoid) supplemented with 10% glycerol and scraping off bacterial growth with a disposable spreader. Cell suspensions were mixed and stored at −80°C. We considered this step as culture-enrichment for *S. pneumoniae* presence.

### Isolation of bacterial DNA from culture-enriched samples

Genomic DNA was purified from cells using the DNeasy Tissue kit (QIAGEN, Venlo, the Netherlands) according to a modified protocol: 200 µl of thawed, mixed sample was transferred to an Eppendorf tube and centrifuged for 2 minutes at 13,000× g. The pellet was re-suspended in 90 µl of 20 mM Tris-Cl, 2 mM EDTA; incubated for 15 minutes at 95°C to inactivate DNAses; and supplemented with 90 µl of 2.4% Triton X-100, 40 mg/ml lysozyme in 20 mM Tris-Cl, 2 mM EDTA to begin cell lysis. Centrifugation was repeated and the supernatant was processed according to the kit's original protocol. DNA was eluted from the columns with 200 µl of elution buffer and stored at 4°C.

### Real-Time PCR targeting *lytA* and *piaA*

Detection of *S. pneumoniae*-specific DNA was conducted by qPCR using primers and probe described by Carvalho *et al*. [21] specific for the gene coding for the major *S. pneumoniae* autolysin LytA and primers and probe specific for the iron uptake ABC transporter lipoprotein PiaA gene [26]: PiaF 5′-CATTGGTGGCTTAGTA AGTGCAA-3′, PiaR 5′-TACTAACACAAGTTCCTGATAAGGCAAGT-3′ and PiaP (probe) 5′-FAM-TGTAAGCGGAAAAGCAGGCCTTACCC-3′-BHQ1. The assays were carried out in 25 µl reaction volumes using TaqMan Universal Master Mix (Life Technologies, Bleiswijk, the Netherlands), 2.5 µl of genomic DNA as a template plus primers at 150 nM and probes for LytA and PiaA at concentrations of 75 nM and 175 nM, respectively. In every run genomic DNA of *S. pneumoniae* strain TIGR4 was used as a positive control [27]. qPCRs were carried out on a StepOnePlus real-time PCR unit (Life Technologies) under the following conditions: 95°C for 10 minutes, followed by 45 cycles of 95°C for 15 s and 60°C for 1 min. Samples were classified as positive for *S. pneumoniae* when C*_T_* values for both targeted genes were below 35 [23], [28].

### Molecular quantification of bacteria

The total bacterial load per sample was determined on DNA templates purified with an Agowa kit (LGC Genomics, Berlin, Germany) using 16S-based qPCR [29] as previously described [30].

### Recovery of live *S. pneumoniae* from culture harvests

Enriched culture harvests from individuals initially classified as not colonized by the conventional culture approach, yet generating any signal specific for *S. pneumoniae* in qPCR were processed in a second attempt to recover live pneumococci. This step was undertaken in order to test whether higher sensitivity instead of false-positivity of qPCR could explain the increased rate of pneumococci detection in adults. In short, 100 µl of 10×, 100×, and 1000× dilutions of thawed culture-enriched samples were spread over the surface of fresh gentamicin plates, incubated for up to 48 hours at 37°C and 5% CO_2_, and screened daily for the presence of pneumococcus-like colonies. *S. pneumoniae* identification was performed by conventional diagnostic approach.

### MLST analysis

Multi Locus Sequence Typing (MLST) was performed as described by Enright and Spratt [31]. The assignment of sequence types (STs) was carried out using online software at the pneumococcal web page: www.mlst.net.

### Statistical analysis

Data were analysed using GraphPad Prism version 5.00 for Windows, (GraphPad Software, San Diego, CA). P-values were calculated by Fisher's exact test.

## Results

We aimed to optimize the method for detection of pneumococcal colonization in nasopharyngeal samples from adults. For this purpose, we compared conventional culturing versus qPCR-based *S. pneumoniae* detection in culture-enriched samples. We tested trans-nasally and trans-orally obtained nasopharyngeal specimens collected from 268 parents of young children, since we expected this adult population to be particularly prone to pneumococcal exposure. Samples analyzed here were a subset of samples collected from 326 parents screened for *S. pneumoniae* carriage in the study published by Spijkerman *et al*. [24]. We included all individuals with both trans-nasal and trans-oral sample cultures available.

First, we cultured matching trans-nasal and trans-oral nasopharyngeal samples from all 268 parents on gentamicin plates and determined the presence of pneumococci by conventional culture according to the procedure recommended by WHO [14]. Next, we isolated DNA from gentamicin plate cultures (culture-enriched samples) and tested for the presence of pneumococci by qPCR. Culture-enriched samples positive for pneumococcus by qPCR yet considered negative at the routine diagnostic culture step were re-cultured and carefully examined for the presence of live *S. pneumoniae*. Figure S1 depicts flow diagram of samples processing in the study.

### Detection of *S. pneumoniae* colonization by routine diagnostic culture approach

Of 268 parents, 52 (19%) were identified as colonized with *S. pneumoniae* at the primary culture step. There were significantly more trans-nasal (n = 49; 18%) compared to trans-oral samples (n = 10; 4%) positive for pneumococci (p<0.001) with seven (3%) individuals culture-positive in both specimens (Table 1). Differences between fractions of parents identified as colonized with *S. pneumoniae* in the original study reported by Spijkerman *et al*. [24] and the subset of individuals analyzed here were not significant (Table S1).

**Table 1:** *Streptococcus pneumoniae* detection in nasopharyngeal samples collected from 268 parents of 24-month-old children according to conventional culture approach and qPCR-based detection of pneumococci in culture-enriched settings.

	Parents classified as positive for *S. pneumoniae*		
Settings	Conventional culture (%) a	qPCR (%) b	Concordance c
Trans-nasal	49 (18)	50 (19)	0.98
Trans-oral	10 (4)	94 (35)	0.68
Both trans-nasal and trans-oral	7 (3)	39 (15)	NA
Either trans-nasal or trans-oral	52 (19)	105 (39)	NA

a Results of conventional culture at the primary diagnostic step;

b Culture harvest templates positive in both *lytA* and *piaA* specific qPCRs with signal below the arbitrary threshold of 35 C*_T_*;

c Degree of similarity between conventional culture and qPCR results with respect to sample identification as positive or negative for *S. pneumoniae*;

NA–not applicable.

### Bacterial density in trans-nasal versus trans-oral cultures

Another notable difference between the trans-nasal and trans-oral cultures from adults was the much lower density of bacterial growth from the trans-nasal compared to the trans-oral swabs, confirmed by bacterial DNA load, with a large fraction of the trans-nasal samples being negative for any alpha-hemolytic growth. Of 268 gentamicin plate cultures of trans-nasal swabs, 159 (59%) were harvested in the study. The remaining 109 (41%) plates showed limited to no bacterial growth, i.e. less than ten colonies, with no alpha-hemolytic colonies present. Since chances that pneumococci would have stayed unnoticed in these 109 cultures seemed negligible, they were not further processed. In contrast, all 268 plates from trans-oral swabs yielded relatively high density bacterial growth.

**Figure 1:**
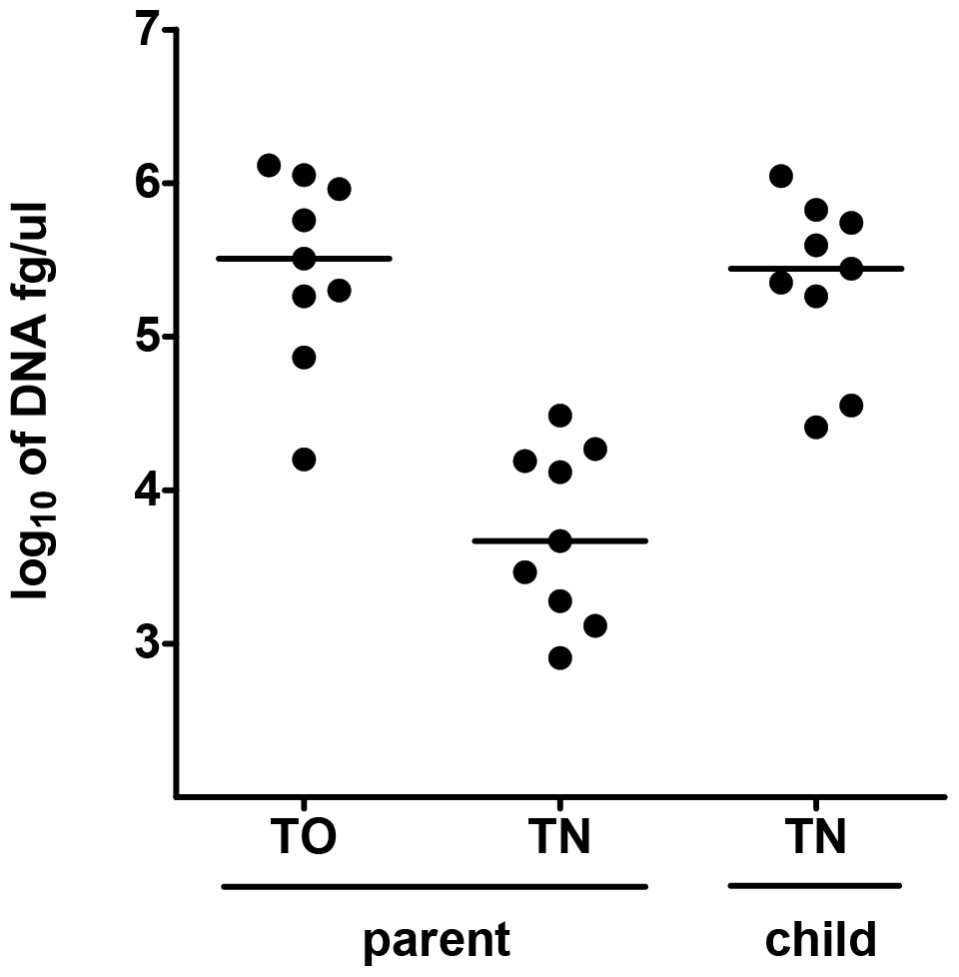
Molecular quantification of bacterial presence in nasopharyngeal samples from children and their parents. Quantity of bacterial DNA in femtograms per microliter of template recovered directly from Amies medium of nasopharyngeal trans-nasal (TN) and trans-oral (TO) samples collected from nine randomly selected parents, plus trans-nasal samples from their children. Horizontal lines represent median concentrations per niche sampled.

This higher yield of bacterial growth from trans-oral samples compared to trans-nasal swabs was not limited to flora selectively growing on gentamicin plates, but was representative of the overall bacteria load in the original specimens. As shown in Figure 1, the qPCR based quantification of the total bacterial DNA in liquid Amies medium samples revealed on average 2log_10_ fewer bacteria in the parental trans-nasal compared to trans-oral specimens. Next, we compared DNA loads of parental samples with those from their children. Samples had been collected in sets of child and parent in this study (parent had both trans-nasal and trans-oral samples analyzed, child only a trans-nasal sample collected and tested). Trans-oral samples in parents showed similar bacterial yields as trans-nasal samples obtained from the children.

### Molecular detection of *S. pneumoniae* in culture-enriched samples

Fifty (19%) culture-enriched trans-nasal samples were classified as positive for *S. pneumoniae* by qPCR (Table 1). These included all but two of the 49 cultures (96%) that were found positive for pneumococcus by initial culture and an additional three from parents that had been classified as non-colonized by the initial culture. For statistical purposes, when analyzing study results we considered the 109 gentamicin plate cultures negative for growth of pneumococcus-like colonies as samples negative for *S. pneumoniae* by qPCR.

All 268 trans-oral cultures showed bacterial growth and were harvested. Of these 268 culture-enriched samples 94 (35%) were classified as positive for *S. pneumoniae* by qPCR, including 9 out of the 10 trans-oral samples that were positive by conventional culture, plus an additional 85 trans-oral samples that were culture-negative in the initial diagnostic step. These included 55 (26%) of 216 trans-oral samples from parents classified as not colonized (culture-negative in both samples). Overall 105 (39%) parents were identified as carriers by the molecular method with 39 (15%) positive in both trans-nasal and trans-oral settings. Opposite to the outcome of the conventional diagnostic step, there were significantly more trans-oral compared to trans-nasal cultures positive for pneumococci by qPCR (94 versus 50; p<0.001).

### Recovery of live *S. pneumoniae* from samples negative for pneumococci by conventional culture yet generating a pneumococcus-specific signal in qPCR

**Figure 2:**
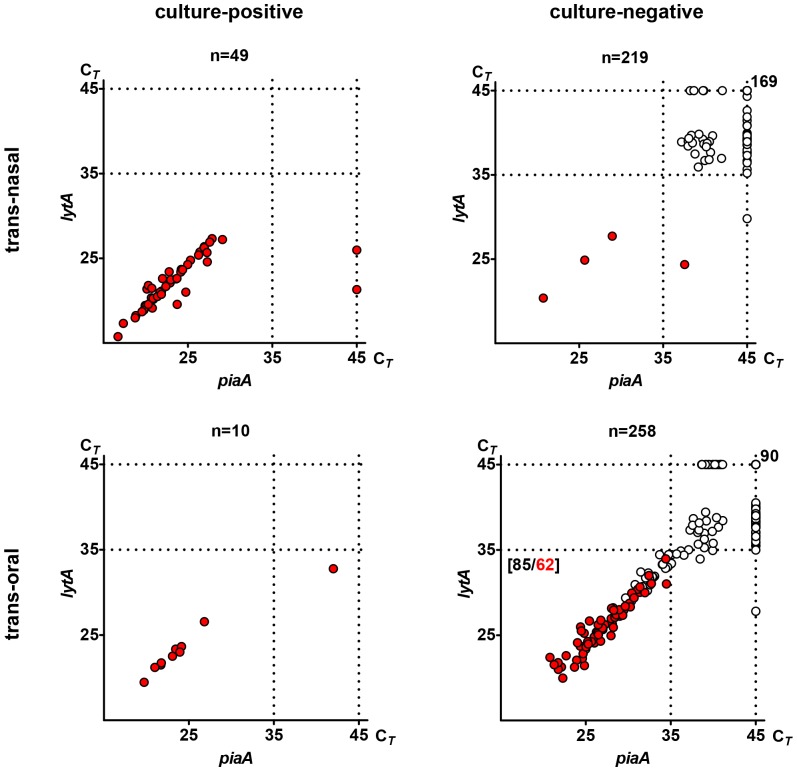
PCR based detection of *Streptococcus pneumoniae* versus recovery of live pneumococci from nasopharyngeal cultures. Figure depicts results of qPCR-based detection of *S. pneumoniae-*specific genes *lytA* and *piaA* and results of live *Streptococcus pneumoniae* isolation from trans-nasal (upper graphs) and trans-oral (lower graphs) nasopharyngeal cultures from 268 parents of 24-month-old children. Samples are divided into culture-positive (graphs on the left) versus culture-negative (graphs on the right) according to the results of conventional culture at the diagnostic step (n  =  number of samples per category). Each dot represents an individual sample. Position of the dot corresponds to C*_T_* values for *lytA-* and *piaA*-specific signals as marked on corresponding axes. Red dots represent samples from which live pneumococci were isolated either from a clinical specimen at the primary diagnostic step (graphs on the left) or from re-processed gentamicin plate culture harvests (graphs on the right). Dotted lines mark the threshold arbitrarily assigned to discriminate between positive (C*_T_*<35) and negative samples, and the total number of 45 cycles in each qPCR reaction. Number next to a dot depicts number of samples with a negative C*_T_* score of 45 for both genes targeted or with limited or no bacterial growth and no growth of alpha-hemolytic colonies in gentamicin plate cultures. Numbers in square brackets depict the number (in black) of samples with C*_T_* values below 35 for both targeted genes/number (in red) of cultures from which live *S. pneumoniae* was isolated at recovery culture step.

Fifty culture-enriched trans-nasal and 168 culture-enriched trans-oral samples classified as negative for *S. pneumoniae* at the primary culture step but positive for any signal (C*_T_* <45) for either of two targeted genes in qPCR were re-cultured in a second attempt to recover live *S. pneumoniae*. Pneumococci were cultured from an additional 4 trans-nasal and 62 trans-oral samples (Figure 2). The latter included 20 trans-oral samples from parents that were already identified as colonized based on isolation of live *S. pneumoniae* from the primary trans-nasal culture. Additionally, live pneumococci were recovered from 43 parents classified as non-colonized at the conventional diagnostic step; pneumococci were isolated either from trans-oral (n = 39), trans-nasal (n = 1), or both samples (n = 3). It increased the overall number of culture-positive individuals to 95 (35%) and individuals culture- and qPCR-positive to 93 (35% of 268). Of those, significantly more were culture- and qPCR-positive in trans-oral (n = 71; 27%) than trans-nasal (n = 50; 19%) samples (p<0.05). The number of parents either culture- or qPCR-positive in the study reached 107 (40%).

Sixty-five of 66 culture harvests from which pneumococci were recovered at this step were positive for *S. pneumoniae* according to the study criteria for qPCR results interpretation, which implies high sensitivity of the qPCR method. After including results of the recovery culture step in results analysis, the concordance between molecular detection and isolation of live pneumococci in trans-oral samples reached 91% (244 of 268), a significant increase (p<0.001) when compared to 68% concordance observed between qPCR and conventional culture results of the diagnostic step alone.

### Sensitivity of methods used for *S. pneumoniae* detection in the study

To assess the sensitivity of methods used in pneumococcal carriage detection in this study we considered all 107 individuals culture- or qPCR-positive in any sample tested as carriers. The molecular detection of pneumococci in trans-oral culture-enriched nasopharyngeal samples show highest sensitivity among methods used (Table 2).

**Table 2:** Sensitivity of methods used to detected *Streptococcus pneumoniae* in nasopharyngeal samples form adults.

*S. pneumoniae* detection method	Sample	Carriers detected	Sensitivity of method a
Culture at the primary diagnostic step	Trans-nasal	49	0.46
	Trans-oral	10	0.09
Molecular method after culture-enrichment	Trans-nasal	50	0.47
	Trans-oral	94	0.88
Culture at either primary diagnostic or recovery step	Trans-nasal	53	0.50
	Trans-oral	72	0.67

a Fraction of 107 individuals identified by any method in the study as positive for *S*. *pneumoniae*.

### The *piaA*-negative strains as a source of discordance between conventional culture and qPCR results

Four culture harvests from three individuals failed to be classified as positive for *S. pneumoniae* by molecular method despite isolation of live *S. pneumoniae* either at the diagnostic or at the recovery steps (Figure 2). In all four cultures *lytA-* but not *piaA*-specific signals were detected below 35 C*_T_*. Optochine-susceptible strains isolated from those samples were negative for *piaA* when tested individually. In order to have further proof those strains were of *S. pneumoniae* species multi-locus sequence types were determined for those isolates [32] MLST confirmed that all four were either of known pneumococcal STs (ST449 and ST393) or single locus variant of ST3691 (www.mlst.net) [31].

## Discussion

The reliable identification of *S. pneumoniae* in the upper airways is a critical bottleneck in colonization studies. Here we used both conventional culture and molecular techniques to detect pneumococci in a population of adults with supposedly greater exposure to the pathogen through family contacts, namely, parents of 24-month old children. The goal was to optimize detection of pneumococcal colonization for future carriage studies in adults. Of note, all children were immunized at 2, 3, 4 and 11 months of age with the heptavalent conjugated polysaccharide pneumococcal vaccine in the Dutch national children immunization program.

By applying the protocol as proposed in this study, we detected the presence of *S. pneumoniae* in an additional quarter of parents that had originally been classified as not colonized by conventional culturing of either trans-nasally or trans-orally derived nasopharyngeal swabs. As a result, the frequency of detected pneumococcal carriage approximately doubled from 19% to 40%. We could attribute this outcome almost entirely to the enhanced detection of *S. pneumoniae* by qPCR in the highly polymicrobial trans-orally obtained nasopharyngeal specimens. Part of this enhanced detection might be because of the culture-enrichment step introduced by us to increase pathogen load and to limit interference due to signal originating from nonviable bacteria, therefore increasing specificity of the molecular detection method [28].

There are a number of published protocols successfully increasing the sensitivity of *S. pnemoniae* detection by culture-enrichment [15], [22], [23]. We collected all growth from cultures on gentamicin plates only after all steps of conventional diagnostic procedure were completed, in order to not interfere with the WHO recommended procedure used in the diagnostic lab. The conventional diagnostic procedure was performed in an experienced reference laboratory that had proved to be highly efficient in the detection of pneumococcal carriage in previous vaccination and surveillance studies [17], [18]. All samples culture-positive for *S. pneumoniae* at the primary diagnostic step were positive for *lytA* in qPCR thus sensitivity of the method was not inferior to WHO recommended protocol. Using published criteria for classifying samples as positive based on the presence of *S. pneumoniae*-specific molecular signal [21], [28] the rate of pneumococcal carriage in our study population reached 40%. It suggests high sensitivity of the carriage detection method we used. By using a labor-intensive culture method, we were also able to isolate live pneumococcus from an extra 16% of parents all classified at the beginning of the study as non-carriers. It increased the number of individuals culture-positive for *S. pneumoniae* from 19% to 35%. Finally, the overall number of samples from which live *S. pneumoniae* was isolated, more than doubled in the study from 59 to 125. These results support high specificity of our method. The fact that pneumococci were recovered after re-culturing from the majority of samples that had been initially identified as culture-negative for the pathogen but were then classified positive for *S. pneumoniae* by qPCR upholds the validity of the qPCR approach.

In our opinion the discordance between conventional diagnostic results and results of molecular detection of *S. pneumoniae* in culture-enriched samples reflects limitations of the routine diagnostic method when trans-oral samples are analyzed. Lower rates of *S. pneumoniae* detection in trans-oral nasopharyngeal or oropharyngeal samples compared to trans-nasal nasopharyngeal samples in adults were reported in pneumococcal surveillance studies published by us and others [17], [19], [20]. As in our present study, they also reported high discordance between conventional culture results for matching trans-nasal and trans-oral samples. Those particular similarities among studies conducted in independent centers seem to support the claim that trans-oral samples are intrinsically difficult to process for *S. pneumoniae*.

The total rate of pneumococcal carriage of 40% observed in this study is one of the highest reported in recent decades for any healthy adult population [10], [18]. Yet we find these results in line with recent reports on *S. pneumoniae* carriage detection in children, where application of molecular methods alone or in combination with culture-enrichment steps significantly increased the number of individuals identified as colonized with pneumococci [23], [33]. It is also in line with historical records of 45% to 60% colonization rates detected in healthy adult persons in the pre-antibiotic era, reported in studies testing oral samples with the sensitive mouse inoculation method [34]. Interestingly, in the original study Spijkerman *et al*. [24] reported rates of pneumococcal carriage in 24-months old children and their parents to match pre-PCV-7 rates of *S. pneumoniae* carriage. This suggests to us that similar high frequency of pneumococcal carriage could be expected among parents of young children independent of child immunization status.

The protocol we applied to determine the presence of *S. pneumoniae* in specimens collected from the upper respiratory tract proved robust and showed no effect of extended storage of the cultures prior to plate harvest (data not shown). We arbitrarily applied relatively stringent criteria to classify a sample as positive for pneumococcus by qPCR: a positive signal for both pneumococcus-specific genes tested, i.e. C*_T_* values below the threshold of 35. Of these, *lytA* is already accepted as a marker for *S. pneumoniae* [21], [35]. Highly conserved *piaA* was chosen as a countermark of pneumococcal DNA presence to uphold the specificity of the qPCR assay in anticipation of possible similarity between *lytA* and its homolog present in other streptococci of the *Streptococcus mitis* group, who share the ecological niche with *S. pneumoniae* [36]–[38]. We found the *lytA*-specific qPCR more sensitive than the molecular assay targeting *piaA*. In concordance with reports published by others, [39], [40] a relatively small fraction of pneumococcal strains were negative for *piaA* in our study. In general however, there was good correlation between qPCRs results for targeted genes when signals at C*_T_*<35 were considered as positive. Discordance between *lytA* and *piaA* qPCR results, as well as a failure to recover live *S. pneumoniae* in both culture steps from samples above this threshold for both genes supports the cut-off for positivity chosen.

The limitation of this study is the cross-sectional nature, since positive qPCR results could reflect in some parents a transient presence of *S. pneumoniae* instead of true colonization. A solution to this would be repeated sampling. Our qPCR data show a high rate of *S. pneumoniae* presence in trans-oral nasopharyngeal samples. We also observed that trans-oral sampling is better tolerated by adults compared to trans-nasal approach. For this reason, when qPCR is used in carriage studies in adults, one could consider processing only trans-oral samples. Repeated sampling should prove whether this is a rapidly transient pathogen presence or if a more stable pneumococcal colonization is observed.

The study strength is enhanced insight into adult human host colonization. Applying similar molecular approaches to determine serotype composition and the presence of other pneumococcal virulence markers could greatly advance our understanding of *S. pneumoniae* circulation in the human population.

In summary, the routine culture was very sensitive in detecting *S. pneumoniae* in trans-nasal nasopharyngeal samples from adults. Neither culture-enrichment nor molecular methods significantly improved detection of *S. pneumoniae* in trans-nasal samples. The sensitivity of a routine diagnostic approach was low when trans-oral samples were tested. The detection of a pneumococcal presence in trans-oral samples dramatically increased when culture-enriched samples were analyzed with molecular method. Overall, *S. pneumoniae* colonization rates detected in our study were significantly higher when trans-oral nasopharyngeal compared to trans-nasal nasopharyngeal samples were tested. Our results argue in favor of trans-oral over trans-nasal nasopharyngeal swabs when evaluating pneumococcal colonization in adults. Further work is needed to develop selective media best suited for enrichment steps for processing samples collected trans-orally. The design should take into consideration the unique ecology of the oral cavity, including higher diversity and higher density of microorganisms present [30]. By applying molecular methods to detect *S. pneumoniae* presences in culture-enriched samples we may gain a better insight into the relatively poorly explored reservoir of pneumococci in the adult human population.

## Supporting Information

Figure S1 Flow chart depicting samples processing in the study.
(TIF)

Table S1 Conventional culture based detection of *Streptococcus pneumoniae* in nasopharyngeal samples collected from parents in the original study published by Spijkerman *et al*. [24] and in the subset analyzed in this study.
(DOC)
