# Supplementary material for: Superiority of Trans-Oral over Trans-Nasal Sampling in Detecting Streptococcus pneumoniae Colonization in Adults
Source: PLoS One. 2013 Mar 28;8(3):e60520. doi: 10.1371/journal.pone.0060520 (PMC3610877; doi:10.1371/journal.pone.0060520)
Supplement: Table S1 — Conventional culture based detection of Streptococcus pneumoniae in nasopharyngeal samples collected from parents in the original study published by Spijkerman et al. [24] and in the subset analyzed in this study. (DOC) [file pone.0060520.s002.doc]

**TABLE S1.** Conventional culture based detection of *Streptococcus pneumoniae* in nasopharyngeal samples collected from parents in the original study published by Spijkerman *et* *al*. and in the subset analyzed in this study.

|  | **Individuals classified as positive for *S. pneumoniae* (%)** | |  |
| --- | --- | --- | --- |
| **Settings** | **Among 326 parents in the study by Spijkerman *et al.*** | **Among 268 parents in this study** | ***P* value ^a^** |
| Either trans-nasal or trans-oral | 66 (20) | 52 (19) | 0.8366 |
| Only trans-nasal | 62 (19) | 49 (18) | 0.8333 |
| Only trans-oral | 12 (4) | 10 (4) | 1.0000 |
| Both trans-nasal and trans-oral | 9 (3) | 7 (3) | 1.0000 |

^a^ Two-tailed Fisher exact probability test.

Reference: Spijkerman J, Prevaes SM, van Gils EJ, Veenhoven RH, Bruin JP *et al.* (2012) Long-term effects of pneumococcal conjugate vaccine on nasopharyngeal carriage of *S. pneumoniae*, *S. aureus*, *H. influenzae* and *M. catarrhalis*. PLoS One 7(6): e39730.
